# Receptor for Hyaluronan Mediated Motility (RHAMM)/Hyaluronan Axis in Breast Cancer Chemoresistance

**DOI:** 10.3390/cancers16213600

**Published:** 2024-10-25

**Authors:** Shiori Fujisawa, Kiyoshi Takagi, Mio Yamaguchi-Tanaka, Ai Sato, Yasuhiro Miki, Minoru Miyashita, Hiroshi Tada, Takanori Ishida, Takashi Suzuki

**Affiliations:** 1 Department of Breast and Endocrine Surgical Oncology, Graduate School of Medicine, Tohoku University, 1-1 Seiryo-machi, Aoba-ku, Sendai 980-8575, Miyagi, Japan; fujisawa.shiori.p3@dc.tohoku.ac.jp (S.F.); minoru.miyashita.c6@tohoku.ac.jp (M.M.); hiroshi.tada.a4@tohoku.ac.jp (H.T.); 2 Department of Pathology and Histotechnology, Graduate School of Medicine, Tohoku University, 2-1 Seiryo-machi, Aoba-ku, Sendai 980-8575, Miyagi, Japan; mio.tanaka.e5@tohoku.ac.jp (M.Y.-T.); ai.sato.b7@tohoku.ac.jp (A.S.); takashi.suzuki.c1@tohoku.ac.jp (T.S.); 3 Personalized Medicine Center, Tohoku University Hospital, Sendai 980-8574, Miyagi, Japan; 4 Department of Anatomic Pathology, Graduate School of Medicine, Tohoku University, Sendai 980-8575, Miyagi, Japan; miki@patholo2.med.tohoku.ac.jp; 5 Department of Pathology, Tohoku University Hospital, Sendai 980-8574, Miyagi, Japan

**Keywords:** RHAMM, hyaluronan, breast cancer, chemoresistance, prognosis

## Abstract

**Background/Objectives**: Receptor for hyaluronan-mediated motility (RHAMM) is a hyaluronan (HA) receptor, which exerts diverse biological functions in not only physiological but also pathological conditions in human malignancies, including breast cancer. Although chemoresistance is a significant clinical challenge in breast cancer, a possible contribution of RHAMM and hyaluronan to breast cancer chemoresistance has remained unclear. **Methods**: We immunolocalized RHAMM and HA in breast carcinoma tissues. Also, we utilized epirubicin-sensitive (parental) and rpirubicin-resistant (EPIR) breast cancer cell lines to explore the role of RHAMMM in breast cancer progression. **Results**: We found out that RHAMM and HA were cooperatively correlated with breast cancer aggressiveness and recurrence after chemotherapy. In vitro studies demonstrated that RHAMM was overexpressed in EPIR cells compared to parental cells. In addition, the knockdown of RHAMM significantly suppressed proliferation and migration of both parental and EPIR cells. On the other hand, the expression level of cancer stem cell marker CD44, which was overexpressed in M-EPIR (epirubicin-resistant MCF-7 subline) compared to MCF-7, was significantly suppressed by knockdown of RHAMM. In addition, the knockdown of RHAMM significantly altered the expression of N-cadherin and E-cadherin, leading to an epithelial phenotype. **Conclusions**: Aberrant RHAMM signaling were considered to cause chemoresistance related to cancer stemness and epithelial to mesenchymal transition, and increased cell proliferation and migration of both chemo-sensitive and chemo-resistant breast cancer cells.

## Simple Summary

Receptor for hyaluronan-mediated motility (RHAMM) is a hyaluronan receptor involved in various functions, including in breast cancer. Chemoresistance in breast cancer is a major clinical issue, and the role of RHAMM and hyaluronan in this process is not well understood. Our study found that RHAMM and hyaluronan are linked to increased aggressiveness and recurrence in breast cancer after chemotherapy. In lab experiments, epirubicin-resistant breast cancer cells had higher RHAMM levels than non-resistant cells. Knockdown of RHAMM decreased the growth and migration of both cell types. It also lowered CD44 levels, a marker of cancer stem cells, and changed N-cadherin and E-cadherin, resulting in more typical epithelial cell behavior. These findings suggest that abnormal RHAMM activity contributes to chemoresistance by enhancing cancer stem cell properties and altering cell characteristics, promoting cancer growth and spread. This research could lead to new ways to combat breast cancer chemoresistance.

## 1. Introduction

Breast cancer is one of the most common malignancies and a leading cause of cancer mortality in women [1]. Although anti-estrogen therapy has significantly improved clinical outcomes of estrogen-dependent breast cancer patients, cytotoxic chemotherapy is still an important therapeutic option for patients with an aggressive phenotype of breast cancer, such as triple-negative breast cancer (TNBC), which lacks hormone both hormone receptors and human epidermal growth factor receptor 2 (HER2). Nevertheless, approximately 25% of breast cancer patients are reported to experience distant metastasis after the chemotherapy [2]. Therefore, it is urgently required to unveil the mechanisms of chemoresistance of breast cancer.

Tumor microenvironment, which consists of not only cellular components but also extracellular matrix (ECM), has recently been implicated in the chemoresistance of human malignancies [3]. Hyaluronan or hyaluronic acid (HA) is a major component of ECM and produced by both tumor cells and stromal cells [4], contributing to the progress of breast cancer [5]. While HA is mainly synthesized by hyaluronan synthase 2 (HAS2), it is digested by several hyaluronidases, resulting in the accumulation of HA with a variety of molecular sizes (low molecular weight HA (LMW HA; <10 kDa) to high molecular weight HA (HMW HA; >1000 kDa) [6]. Importantly, the role of HA is highly dependent on its size [7], and LMW HA is considered to promote breast cancer progression, while HMW HA suppresses [6]. In addition, HA is closely related to chemoresistance in human malignancies [8,9].

HA binds to its receptor to transmit intracellular signaling. CD44 and receptor for hyaluronan-mediated motility (RHAMM) are major receptors of HA. While CD44 binds to LMW HA and HMW HA with a similar affinity [6], RHAMM preferentially binds to LMW HA [10], possibly indicating that RHAMM mediates pro-tumorigenic effects of HA. In addition, RHAMM is related to worse clinical outcomes of human malignancies, including endometrial cancer, oral squamous cell carcinoma, prostate cancer [11,12,13], as well as breast cancer [14,15,16]. However, a possible contribution of RHAMM to breast cancer chemoresistance remains to be fully elucidated. Therefore, we conducted an immunohistochemical analysis of RHAMM using breast cancer specimens to address their possible role in breast cancer. We also subsequently examined molecular mechanisms of breast cancer chemoresistance associated with RHAMM using chemo-sensitive/resistant breast cancer cell lines.

## 2. Materials and Methods

### 2.1. Patients and Tissues

114 formalin-fixed paraffin-embedded surgical specimens of invasive breast cancer (no special type) were obtained from patients at Tohoku University Hospital from 2007 to 2008. The fixation was usually completed within 72 h. Clinical outcome was defined by disease-free survival (median (min-max); 58.5 (3–84) months).

The study was reviewed and approved by the Ethics Committee at Tohoku University Graduate School of Medicine.

### 2.2. Immunohistochemistry

Antigen retrieval for RHAMM immunostaining was conducted by autoclave (in Tris-HCl/EDTA buffer (pH 9)). Histofine kit (Nichirei, Tokyo, Japan), which consists of blocking reagent, secondary antibody, and peroxidase-conjugated streptavidin, was used according to manufacturer’s protocol and previous report [17,18]. The antibody against RHAMM was purchased from Novus Biological (Littleton, CO, USA) and used in 1/2000 dilution. Immune complex was visualized by diaminobenzidine and counterstained by hematoxylin. Human stomach was used as a positive control of RHAMM immunostaining.

### 2.3. Scoring of RHAMM Immunoreactivity and Other Histochemical Parameters

RHAMM immunoreactivity was detected in the plasma membrane of the cytoplasm of carcinoma cells, and the cases with reactivity of ≥20% were considered positive [19].

Immunohistochemical status of estrogen receptor (ER), progesterone receptor (PR), HER2, and Ki67 were referred from our previous studies [20], and LI ≥ 1% was considered positive for ER and PR. An intrinsic subtype of breast cancer was defined according to St. Gallen 2011 surrogate definition (luminal A: positive for ER/PR and Ki67 LI < 14%, luminal B: positive for ER/PR, negative for HER2 and Ki67 LI ≥ 14%, and positive for ER/PR and positive for HER2, TNBC: negative for all). In addition, histochemical HA status was referred from another work of ours [21].

### 2.4. Cell Lines and Chemicals

Breast cancer cell lines MCF-7, T-47D, and MDA-MB-231 were purchased from the American Type Culture Collection (Manassas, VA, USA) and cultivated in RPMI-1640 medium (Fujifilm Wako, Osaka, Japan) with 10% fetal bovine serum (Biosera, Nuaillé, France). 4-Methylumbelliferone (4-MU), a HA synthesis inhibitor, was purchased from Tokyo Kasei Kogyo (Tokyo, Japan). Chemotherapeutic agent epirubicin (EPI) was purchased from Fujifilm Wako.

### 2.5. Establishment of EPI-Resistant Breast Cancer Cell Lines

To establish EPI-resistant breast cancer cell lines, MCF-7 and MDA-MB-231 were exposed to increasing doses of EPI (10 nM to 300 nM) for 96 h, followed by recovery culture (without EPI) for 72 h. This cycle continued until MCF-7 and MDA-MB-231 acquired stable growth in 300 nM EPI and then named M-EPIR and 231-EPIR, respectively. M-EPIR and 231-EPIR were cultured with 150 nM EPI to maintain the resistance to EPI.

### 2.6. Real Time PCR

RNA extraction and cDNA synthesis was carried out according to the previous reports [20]. PCR was carried out using the THUNDERBIRD SYBR qPCR Mix (Toyobo, Osaka, Japan) and Light Cycler Nano (Roche Diagnostics Japan, Tokyo, Japan). The sequence of primers was described in Supplementary Table S1. The mRNA expression level of target genes was normalized by *RPL13A* and presented as relative mRNA expression level (%).

### 2.7. Immunoblotting

Protein extraction, SDS-PAGE, and detection of chemiluminescence were according to the previous reports [20]. In the present study, 10 μg of protein lysate was separated by SDS-PAGE, and the RHAMM antibody for immunoblotting was the same as that in immunohistochemistry (1/2000 dilution). β-actin was used as a loading control.

### 2.8. Small Interfering RNA (siRNA) Transfection

Two siRNA targeting RHAMM (siRHAMM-1, 2) and MISSION siRNA Universal Negative Control (siCTRL) were purchased from Ajinomoto Bio-Pharma Services (Osaka, Japan) and Sigma Aldrich (St. Louis, MO, USA), respectively. The sequence of siRHAMM is listed in Supplementary Table S1. Breast cancer cell lines were transfected with these siRNAs (final concentration; 5 nM) using Lipofectamine 3000 transfection reagent (Thermo Fisher Scientific, Waltham, MA, USA).

### 2.9. Cell Proliferation Assay

Each cell line (5 × 10^3^/well) was seeded in a 96-well plate and allowed to attach for 24 h. After the transfection with siRNAs or treatment with 4-MU, proliferation ability was evaluated by Cell Counting Kit-8 (Dojindo Molecular Technologies, Kumamoto, Japan) and presented as relative cell proliferation (%) compared to the control group.

### 2.10. Wound Healing Assay

Migration ability was assessed by wound healing assay using culture inserts (Platypus Technologies, Madison, WI, USA). Briefly, the cells were seeded in a 96-well plate (>90% confluency) and allowed to attach for 24 h. Then, the inserts were removed, and the migrating area was measured using ImageJ 1.52a software (https://imagej.nih.gov/ij/, accessed on 5 June 2024). Cell migration was defined as the ratio of migrating area to an initial gap (0 h) and presented as relative cell migration (%) compared to the control group.

### 2.11. Statistical Analysis

Statistical analyses were performed using JMP Pro Ver 17 (SAS Institute, Cary, NC, USA). $\chi^{2}$ test or Wilcoxon rank sum test was used to examine the correlation between RHAMM status and each clinicopathological parameter. Disease-free survival was expressed by Kaplan-Meier curve and examined by log lank test. Univariate and multivariate analyses were performed using Cox proportional hazard model. Fisher’s Protected Least Significant Difference (PLSD) test was used for the in vitro studies. Statistical significance was defined by *p* < 0.05. In the prognostic analysis using a proportional hazard model, the parameters with *p* < 0.1 in the univariate analysis were also incorporated in subsequent multivariate analysis.

## 3. Results

### 3.1. Immunolocalization of RHAMM in Human Breast Carcinoma Tissues

**Figure 1:**
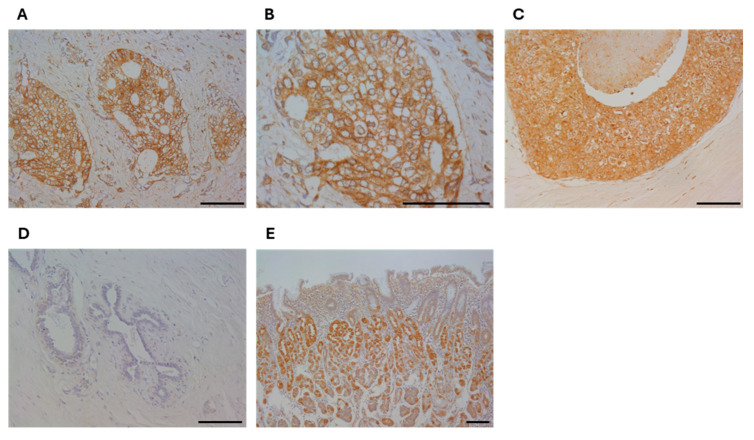
Immunolocalization of RHAMM in human breast carcinoma tissues. Immunoreactivity of breast carcinoma cells ((**A**), ×200, (**B**), ×400, (**C**), in situ component (×200), normal breast epithelium ((**D**), ×200), and positive control; stomach ((**E**), ×100). Bar = 100 μm, respectively.

RHAMM was immunolocalized in the cytoplasm or plasma membrane of breast carcinoma cells (Figure 1A–C). It was also detected in the carcinoma cells of the in situ component (Figure 1C), while it was almost negligible in normal breast epithelium (Figure 1D). In the positive control tissue (human stomach), RHAMM immunoreactivity was detected in the fundic gland (Figure 1E).

### 3.2. Clinical Significance of RHAMM

The correlation between RHAMM status and clinicopathological parameters is summarized in Table 1. RHAMM status was positively correlated with pathological T factor (pT) (*p* < 0.046), lymph node metastasis (*p* = 0.0052), stage (*p* = 0.011), and Ki67 labeling index (*p* = 0.012) as well as HA (*p* = 0.033). Importantly, when the patients were divided into two groups according to HA status, these correlations were maintained only in the HA-positive group but not in the HA-negative group. On the other hand, RHAMM status was not correlated with breast cancer subtypes.

**Figure 2:**
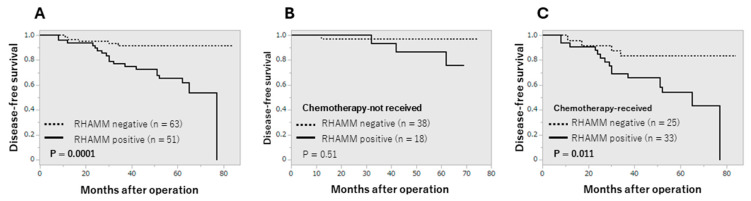
Disease-free survival curve according to RHAMM status. (**A**), All cases (n = 114), (**B**), chemotherapy-not received group (n = 56), (**C**), chemotherapy-received group (n = 58).

The correlation between RHAMM status and patients’ outcomes is summarized in Figure 2. RHAMM status was significantly correlated with an increased risk of recurrence (*p* = 0.0001, Figure 2A). Interestingly, RHAMM status was correlated with worse clinical outcomes in the patients who received chemotherapy but not in those without the therapy (Figure 2B,C). In addition, when we correlated RHAMM status with clinical outcome according to the subtype, RHAMM was correlated with worse clinical outcome in the luminal A group (*p* = 0.024) (Supplementary Figure S1).

**Figure 3:**
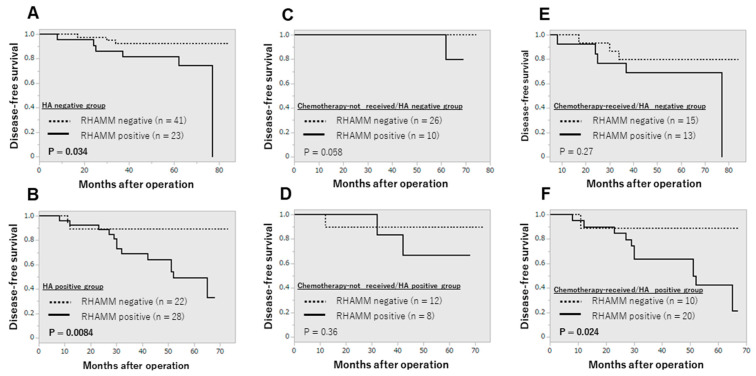
Disease-free survival curve according to RHAMM and HA. (**A**); HA-negative group (n = 64), (**B**); HA-positive group (n = 50), (**C**); chemotherapy-not received/HA-negative group (n = 36), (**D**); chemotherapy-not received/HA-positive group (n = 20), (**E**); chemotherapy-received/HA-negative group (n = 28), (**F**); chemotherapy-receive/HA-positive group (n = 30).

Since we have identified HA as a worse prognostic factor in breast cancer [21], we examined whether RHAMM contributed to the recurrence of breast cancer patients in cooperation with HA or not. As shown in Figure 3, RHAMM status was correlated with increased risk of recurrence only in the HA-positive group (Figure 3A,B). Furthermore, when the patients were divided into four groups according to HA and chemotherapy status, RHAMM status was correlated with recurrence only in the HA-positive/chemotherapy-received group (Figure 3C–F). This tendency was also observed in the luminal A group but not in other subtypes (Supplementary Figure S2).

The prognostic implication of RHAMM was confirmed by uni- and multivariate analysis. As shown in Table 2, RHAMM status turned out to be an independent prognostic factor for the recurrence of breast cancer. This finding was maintained when the patients were limited to those who received chemotherapy (Table 3).

### 3.3. Increased RHAMM Expression in EPI-Resistant Breast Cancer Cell Lines

Since our immunohistochemical analysis demonstrated a close relationship between RHAMM and chemoresistance of breast cancer, we subsequently validated this finding using chemo-resistant breast cancer cell lines (M-EPIR and 231-EPIR) as well as parental chemo-sensitive cells. We used both MCF-7 lineage and MDA-MB-231 lineage because RHAMM status was not correlated with ER.

**Figure 4:**
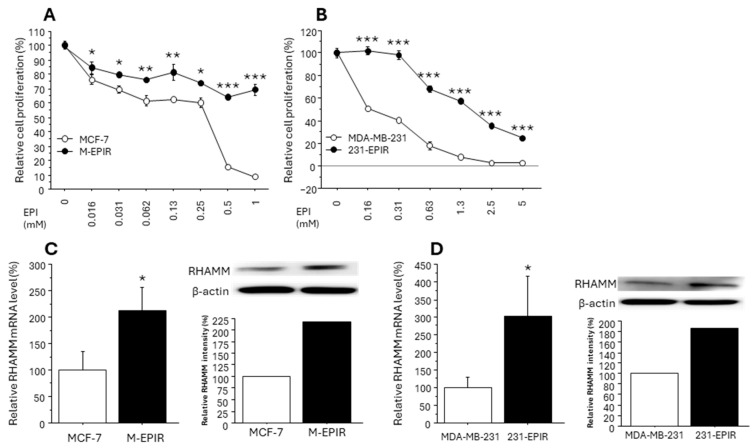
Sensitivity to epirubicin (EPI) and RHAMM expression in chemo-sensitive (MCF-7 and MDA-MB-231) and chemo-resistant cell lines (M-EPIR, 231-EPIR). (**A**,**B**), comparison of the sensitivity to EPI between MCF-7 and M-EPIR (**A**), or MDA-MB-231 and 231-EPIR (**B**) (n = 4). (**C**,**D**), mRNA and protein level of RHAMM in MCF-7 or M-EPIR (**C**) and MDA-MB-231 or 231-EPIR (**D**) (n = 4 for real time PCR). *; *p* < 0.05, **; *p* < 0.01, and ***; *p* < 0.001, respectively. (**C**,**D**); mRNA and protein level of RHAMM in MCF-7 or M-EPIR (**C**), and MDA-MB-231 or 231-EPIR (**D**) (n = 4 for real time PCR). *; *p* < 0.05, **; *p* < 0.01, and ***; *p* < 0.001, respectively. The original Western blot figures can be found in Supplementary File S1.

In the cell proliferation assay performed in advance of functional analysis, we confirmed that both M-EPIR and 231-EPIR had acquired significant resistance to EPI compared to parental cells (Figure 4A,B). We then compared the RHAMM expression between parental cells and EPI-resistant cells. As shown in Figure 4C,D, *RHAMM* mRNA and protein expression were significantly increased in M-EPIR and 231-EPIR cells.

**Figure 5:**
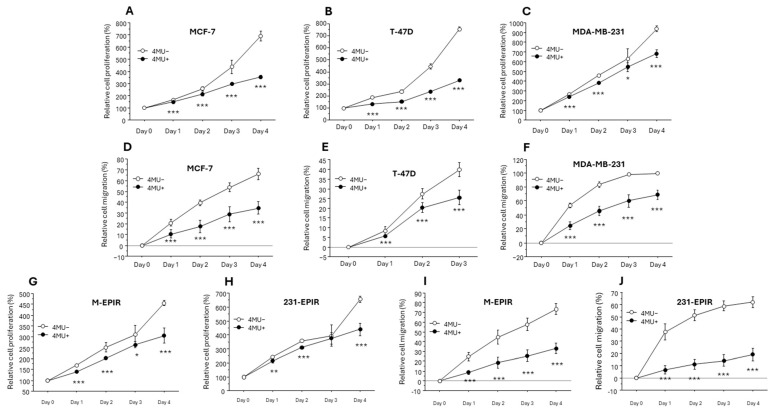
The effect of 4-MU on cell proliferation and migration in chemo-sensitive and chemo-resistant cell lines. (**A**–**F**); cell proliferation ((**A**–**C**), n = 4) and migration ((**D**–**F**), n = 6) in MCF-7 (**A**,**D**), T-47D (**B**,**E**) or MDA-MB-231 (**C**,**F**). (**G**–**J**); cell proliferation (**G**,**H**) and migration (**I**,**J**) in M-EPIR (**G**,**I**) or 231-EPIR (**H**,**J**). *; *p* < 0.05, **; *p* < 0.01 and ***; *p* < 0.001, respectively.

**Figure 6:**
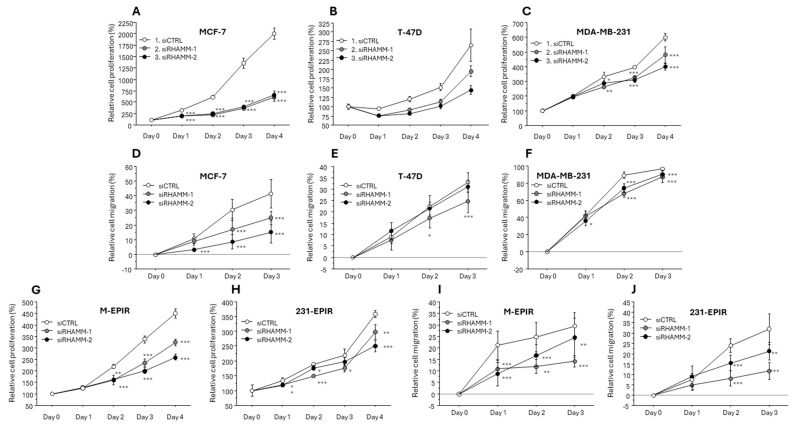
The effect of RHAMM knockdown siRNAs on cell proliferation and migration in chemo-sensitive and chemo-resistant cell lines. Cell proliferation ((**A**–**C**,**G**,**H**), n = 4) and migration ((**D**–**F**,**I**,**J**), n = 6) in MCF-7 (**A**,**C**), T-47D (**B**,**E**), MDA-MB-231 (**C**,**F**), M-EPIR (**G**,**I**), and 231-EPIR (**H**,**J**). *, *p* < 0.05, **; *p* < 0.01, and ***; *p* < 0.001, respectively.

We next examined the effect of HA synthesis inhibitor 4-MU on breast cancer cell proliferation and migration (Figure 5). 4-MU significantly inhibited cell proliferation and migration in both MCF-7, T-47D and MDA-MB-231 (Figure 6A–F). In addition, 4-MU also inhibited cell proliferation and migration of M-EPIR and 231-EPIR (Figure 5G–J).

We subsequently examined the effects of RHAMM on the proliferation and migration of chemo-sensitive and chemo-resistant cells using siRNAs for RHAMM. The knockdown efficiency was confirmed by real time PCR, immunoblotting, or both (Supplementary Figure S3). Knockdown of RHAMM resulted in a significant decrease of both chemo-sensitive cells and chemo-resistant cells of MCF-7 and MDA-MB-231 lineages (Figure 6).

### 3.4. Possible Regulation of Cancer Stem Cell Marker/Another HA Receptor CD44 and EMT-Related Marker by RHAMM

**Figure 7:**
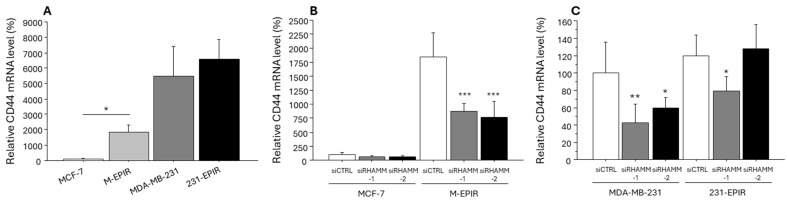
RHAMM expression in chemo-sensitive and chemo-resistant cell lines and the effect of RHAMM knockdown on CD44 expression in breast cancer cell lines. (**A**), comparison between MCF-7 and M-EPIR, or MDA-MB-231 and 231-EPIR, (**B**,**C**), effect of RHAMM knockdown on CD44 expression in MCF-7 lineage (**B**) and MDA-MB-231 lineage (**C**). *; *p* < 0.05, **; *p* < 0.01 and ***; *p* < 0.001 compared to control group.

We examined the effect of RHAMM on the expression of CD44 and EMT-related marker (N-cadherin as a mesenchymal marker and E-cadherin as an epithelial marker). Because CD44 is known as a cancer stem cell (CSC) marker and CSCs are deeply associated with chemoresistance of various human malignancies. As we had expected, the expression of *CD44* mRNA was significantly upregulated in M-EPIR compared to MCF-7, suggesting the increased stemness in M-EPIR (Figure 7A), whereas it was comparable in MDA-MB-231 and 231-EPIR. In addition, *CD44* mRNA was significantly suppressed by the knockdown of RHAMM in M-EPIR (Figure 7B). *CD44* mRNA in MDA-MB-231 was also suppressed by knockdown of RHAMM, while that in 231-EPIR was only partially decreased (Figure 7C). CD44 mRNA was also suppressed by knockdown of RHAMM in T-47D (Supplementary Figure S4).

**Figure 8:**
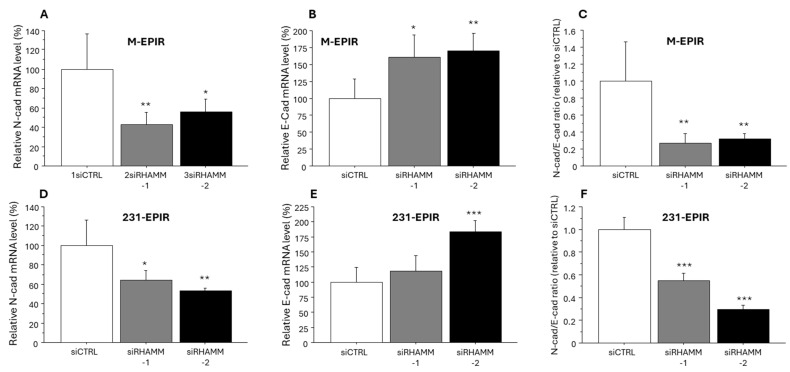
The effect of RHAMM knockdown on the expression of N-Cadherin (mesenchymal marker), E-cadherin (epithelial marker) in breast cancer chemo-resistant cell lines. Relative *N-cadherin* mRNA level (**A**,**D**), relative *E-cadherin* mRNA level (**B**,**E**) and *N-cadherin/E-cadherin* ratio ((**C**,**F**), relative to siCTRL) in M-EPIR (**A**–**C**) and 231-EPIR (**D**–**F**). *; *p* < 0.05, **; *p* < 0.01 and ***; *p* < 0.001.

We finally evaluated the expression level of N-cadherin and E-cadherin to examine the effect of RHAMM on EMT in breast cancer. As shown in Figure 8, expression of *N-cadherin* mRNA was significantly suppressed by knockdown of RHAMM, while that of *E-cadherin* was increased in M-EPIR and 231-EPIR. N-cad/E-cad ratio was also significantly decreased in both EPI-resistant cells.

## 4. Discussion

In the present study, RHAMM status was markedly detected in breast carcinoma cells compared to normal breast epithelium, suggesting that RHAMM has an important role in breast cancer. We demonstrated that RHAMM status was significantly correlated with pT, lymph node metastasis, stage, and Ki67. To the best of our knowledge, this is the first report demonstrating the correlation between RHAMM status and pT or Ki67, important biomarkers of breast cancer [22,23]. In accordance with this finding, we also demonstrated that the knockdown of RHAMM significantly suppressed proliferation and migration in MCF-7, T-47D, and MDA-MB-231 cells. RHAMM is therefore considered to promote proliferation, migration, and metastasis in breast cancer. On the other hand, it has been interestingly reported that RHAMM inhibits migration and lung metastasis of T-47D breast cancer cell line (Luminal A type), while RHAMM promotes migration of MDA-MB-231 (basal type) [24]. In addition, loss of RHAMM promoted lung metastasis of MMTV-PyMT mouse breast cancer model [25]. However, in the present study, we could not detect a significant correlation between RHAMM and breast cancer subtype, and contributions of RHAMM to breast cancer proliferation/migration in each subtype remain unclear, warranting further investigation with larger samples size. On the other hand, the correlation between RHAMM status and aggressive phenotype of breast cancer was detected, especially in the HA-positive group, but not in the HA-negative group. In addition, RHAMM status was positively correlated with that of HA, and this may arise from that HA binds to RHAMM to induce RHAMM expression [26]. Considering our present results and previous findings demonstrating that HA synthesis is increased in breast carcinoma tissues [4,27], RHAMM and HA might cooperatively contribute to the proliferation, invasion, and metastasis of breast cancer.

Similarly, RHAMM status was significantly correlated with an increased risk of recurrence. Importantly, the prognostic significance of RHAMM was prominently found in the patients who received chemotherapy dependent on HA. We also demonstrated that RHAMM status was an independent prognostic factor for disease-free survival of breast cancer, and this is consistent with previous report, in which RHAMM is identified as a worse prognostic factor in breast cancer [12,13,14]. This finding might indicate possible role of HA/RHAMM in the chemoresistance of breast cancer. On the other hand, correlation between RHAMM and breast cancer recurrence was detected in only the luminal A group. Luminal A breast cancers usually show estrogen dependency, and several estrogen-regulated genes are associated with the recurrence of estrogen-dependent breast cancer [28,29]. However, we could not suggest possible regulation of RHAMM by ER since there was no significant correlation between them. In addition, chemotherapy is not frequently applied to luminal A breast cancer. Possible implication of RHAMM in chemoresistance of luminal A breast cancer might warrant further examinations. It has been reported that the pro-proliferative effect of HA in Kv562, a vincristine-resistant human leukemic cell line derived from K562, is mediated by RHAMM, whereas that of parental K562 is mediated by mainly CD44 [30]. We also demonstrated that expression of RHAMM is increased in EPI-resistant cell line M-EPIR and 231-EPIR, and knockdown of RHAMM as well as inhibition of HA synthesis by 4-MU resulted in a decreased cell proliferation of them. In addition, higher serum HA level is associated with shorter progression-free and overall survival of ovarian cancer patients [31]. Similarly, serum HA levels were higher in breast cancer patients who recurred after chemotherapy [32]. Considering these findings from previous reports and our present study, RHAMM can also be considered an important target molecule to overcome the chemoresistance of breast cancer, while degradation of intratumoral HA using PEGylated (polyethylene glycol-attached) recombinant human hyalronidase (PEGPH20) is already considered a potent therapeutic strategy of human cancers [33,34].

To address the possible mechanism of chemoresistance mediated by RHAMM, we examined the expression of CD44, another receptor of HA and representative marker of breast cancer stem cells (CSCs) [35], because CSCs are closely associated with chemoresistance of human malignancies, including breast cancer [36,37]. In the MCF-7 lineage, expression of CD44 was significantly increased following the acquisition of resistance to EPI, suggesting the importance of enriched stemness in our EPI-resistant model. Importantly, the knockdown of RHAMM significantly suppressed the expression of RHAMM in M-EPIR. Therefore, these findings may suggest that the acquisition of chemoresistance by RHAMM is associated with enriched stemness of breast cancer. In addition, RHAMM interacts with CD44 to activate ERK1/2 in breast cancer cell lines [26], and CD44 is reported to upregulate MDR1 microRNA-21 (miR-21), resulting in MDR1 (multi drug resistance protein 1) or anti-apoptotic protein Bcl2 in breast cancer cells [38,39]. Therefore, it is also speculated that RHAMM and CD44 cooperatively contribute to chemoresistance of breast cancer. On the other hand, the expression of CD44 in 231-EPIR is almost comparable to that of parental MDA-MB-231, and the effect of RHAMM knockdown on CD44 mRNA expression was relatively limited. This may partly be due to the fact that basal CD44 expression level is much higher in MDA-MB-231 compared to MCF-7 [40], and other mechanisms may be associated with increased stemness in MDA-MB-231.

In addition to increased stemness, EMT is a pivotal mechanism in the chemoresistance of breast cancer [41,42]. In the present study, we demonstrated that knockdown of RHAMM altered the expression of N-cadherin and E-cadherin, leading to an epithelial phenotype. In concordant with this finding, RHAMM has been reported to promote EMT in A549 lung cancer cell line [43]. In addition, RHAMM has been reported to confer chemoresistance by inducing EMT via TGF*β*/Smad2 in gastric cancer [44]. RHAMM might, therefore, contribute to the chemoresistance of breast cancer by inducing EMT. However, Opposite findings have also been previously reported by Wang et al., in which they demonstrated that RHAMM suppresses EMT in luminal type breast cancer cell lines, MCF-7 and T-47D, whereas RHAMM promotes EMT in basal type breast cancer cell line, MDA-MB-231 [23]. This discrepancy may partly arise from the use of different EMT markers in the experiments. It might be important to examine the role of RHAMM in EMT using multiple epithelial/mesenchymal markers.

As a limitation, we have not confirmed in vitro whether the pro-tumorigenic effects of RHAMM are dependent on HA or not. While we employed 2D culture for in vitro experiments, HA is known to have stronger physiological activities under 3D conditions compared to 2D one [45,46]. Further investigation using xenograft model of organoid model in which RHAMM is stably suppressed might be required. In addition, we could not demonstrate recovery of sensitivity to EPI by knockdown of RHAMM in EPI-resistant model. There might be multiple mechanisms behind the chemoresistance of breast cancer other than RHAMM, while RHAMM immensely contributes to the growth or migration of breast cancer cells which have acquired resistance to chemotherapy. Also, the mechanisms of chemoresistance may differ among the kinds of chemotherapeutic agents, although we have not succeeded in the establishment of other chemo-resistant cell lines than EPI-Rs. Our present study is, therefore, a starting point for exploring of the precise mechanisms of the RHAMM/HA axis in breast cancer progression.

## 5. Conclusions

We demonstrated that RHAMM immensely contributes to breast cancer chemoresistance by several mechanisms: promoting proliferation and migration, enrichment of breast cancer stemness, and induction of EMT. Also, we found that RHAMM served as a potent prognostic factor in breast cancer patients, especially in those who received chemotherapy.

## Figures and Tables

**Table 1:** Clinicopathological characteristics of RHAMM immunoreactivity in breast cancer tissues.

		All Cases			HA Negative			HA Positive		
		RHAMM			RHAMM			RHAMM		
		Negative (n = 63)	Positive (n = 51)	*p*	Negative (n = 41)	Positive (n = 23)	*p*	Negative (n = 22)	Positive (n = 28)	*p*
Age *		57 (27–87)	57 (29–82)	0.93	56 (33–87)	56 (29–82)	0.93	58 (27–77)	57 (40–82)	0.77
Menopause										
	Pre	24	17		15	10	0.59	9	7	0.23
	Post	39	34	0.60	26	13		13	21	
pT										
	pT1	47	29	**0.046**	30	18	0.65	17	11	**0.0072**
	pT2–4	16	22		11	5		5	17	
Lymph node metastasis										
	negative	50	28	**0.0052**	34	16	0.21	16	12	**0.035**
	positive	13	23		7	7		6	16	
Stage										
	1	44	22	**0.011**	29	14	0.11	15	8	**0.020**
	2	10	19		6	8		4	11	
	3	9	10		6	1		3	9	
Histological grade										
	1	30	15	0.064	21	7	0.25	9	8	0.37
	2	24	21		15	11		9	10	
	3	9	15		5	5		4	10	
ER										
	negative	9	13	0.13	6	7	0.13	3	6	0.48
	positive	54	38		35	16		19	22	
PR										
	negative	19	18	0.56	13	9	0.55	6	9	0.71
	positive	44	33		28	14		16	19	
HER2										
	negative	54	43	0.83	34	10	0.67	20	23	0.38
	positive	9	8		7	3		2	5	
Ki67 LI (%) *		9 (1–60)	18 (1–53)	**0.012**	8 (1–60)	7 (1–49)	0.37	10.5 (1–41)	18 (1–53)	**0.019**
Subtype										
	Luminal A	34	19	0.18	23	11	0.095	11	8	0.31
	Luminal B	20	19		12	5		8	14	
	HER2	4	3		4	1		0	2	
	TNBC	5	10		2	6		3	4	
HA										
	negative	41	23							
	positive	22	28	**0.033**						

*; Data were presented as median (minimum–max), and statistical significance was assessed by the Wilcoxon rank sum test. All other values were presented as the number of cases, and statistical significance was assessed by $\chi^{2}$ test. *p* < 0.05 was considered significant and described in bold. ER, estrogen receptor, HER2, human epidermal growth factor receptor 2, LI, labeling index, PR, progesterone receptor, TNBC, triple-negative breast cancer.

**Table 2:** Uni- and multivariate analysis of disease-free survival of breast cancer patients.

		Univariate *		Multivariate	
		*p*	Relative Risk (95% CI)	*p*	Relative Risk (95% CI)
pT	pT2–4/pT1	**0.0002**	5.0 (2.1–12)	0.056	3.3 (0.97–11)
Lymph node metastasis	positive/negative	**0.0024**	3.5 (1.6–8.0)	0.98	0.99 (0.32–3.1)
Histological grade	3/1 + 2	**0.0037**	3.4 (1.5–7.6)	0.18	0.46 (0.15–1.4)
ER	negative/positive	**0.0062**	3.2 (1.4–7.3)	0.61	0.73 (0.21–2.5)
PR	negative/positive	**0.0007**	4.4 (1.9–10)	**0.007**	4.6 (1.5–14)
HER2	positive/negative	0.22	0.41 (0.096–1.7)		
Ki67 LI	≥20%/<20%	**0.0002**	4.8 (2.1–11)	0.097	2.7 (0.83–9.0)
HA	positive/negative	**0.0097**	3.1 (1.3–7.4)	0.22	1.9 (0.67–5.4)
RHAMM	positive/negative	**0.0006**	5.6 (2.1–15)	**0.005**	4.8 (1.6–15)

*, *p* < 0.05 was considered significant (bold) and incorporated in multivariate analysis.

**Table 3:** Uni- and multivariate analysis of disease-free survival of breast cancer patients who received chemotherapy.

		Univariate *		Multivariate	
		*p*	Relative Risk (95% CI)	*p*	Relative Risk (95% CI)
pT	pT2–4/pT1	*0.066*	2.6 (0.94–7.2)	**0.018**	3.9 (1.3–12)
Lymph node metastasis	positive/negative	0.24	1.8 (0.68–4.7)		
Histological grade	3/1 + 2	0.21	1.8 (0.72–4.3)		
ER	negative/positive	0.23	0.58 (024–1.4)		
PR	negative/positive	0.056	2.7 (0.98–7.5)	**0.009**	4.8 (1.5–15)
HER2	positive/negative	*0.077*	0.27 (0.062–1.2)	**0.017**	0.16 (0.035–0.72)
Ki67 LI	≥20%/<20%	0.044	2.6 (1.0–6.6)	0.63	1.3 (0.44–3.9)
HA	positive/negative	0.20	1.8 (0.72–4.7)		
RHAMM	positive/negative	0.018	3.8 (1.3–11)	**0.01**	4.4 (1.4–14)

*, *p* < 0.05 was considered significant (bold), and 0.05 ≤ *p* < 0.1 was considered border line significant (italic), respectively. The parameters with *p* < 0.1 were incorporated in multivariate analysis.

## Data Availability

All data and materials presented in this article and in the Supplementary Documents are available from the corresponding author on reasonable request.

## Supplementary Materials

The following supporting information can be downloaded at: https://www.mdpi.com/article/10.3390/cancers16213600/s1, File S1: The original Western blot figures; Figure S1: Disease-free survival according to RHAMM in each breast cancer subtype; Figure S2: Disease-free survival according to RHAMM and HA in each breast cancer subtype, Figure S3: Knockdown efficiency of siRNA targeting RHAMM (siRHAMM-1 and siRHAMM-2); Figure S4: Regulation of CD44 mRNA by RHAMM in T-47D; Table S1: The sequence primers and siRNAs.
