# Supplementary material for: Receptor for Hyaluronan Mediated Motility (RHAMM)/Hyaluronan Axis in Breast Cancer Chemoresistance
Source: Cancers (Basel). 2024 Oct 25;16(21):3600. doi: 10.3390/cancers16213600 (PMC11545538; doi:10.3390/cancers16213600)
Supplement: Supplementary file 1 [file cancers-16-03600-s001.zip › cancers-3265332-supplementary.pdf]

Supplementary Figure S1

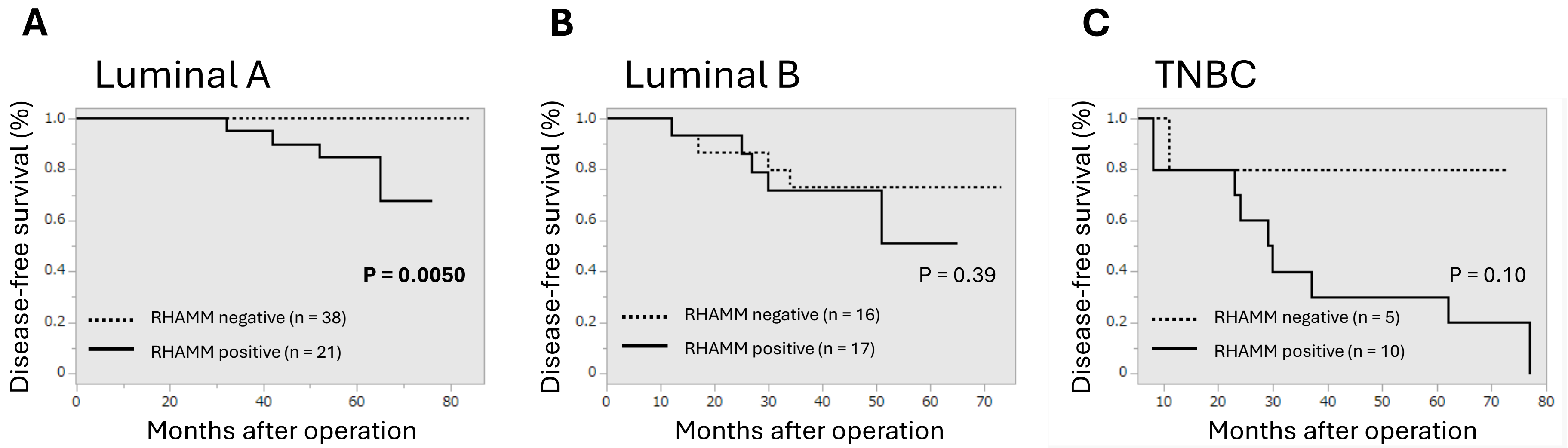

Supplementary Figure S1. Disease-free survival according to RHAMM in each breast cancer subtype

Survival curves in luminal A (A), luminal B (B) and TNBC subtype (C) were generated by Kaplan-Meier method and statistical significance was assessed by log lank test.

Survival curve of HER2 type was not available because no patients had recurred in this group.

Supplementary Figure S2

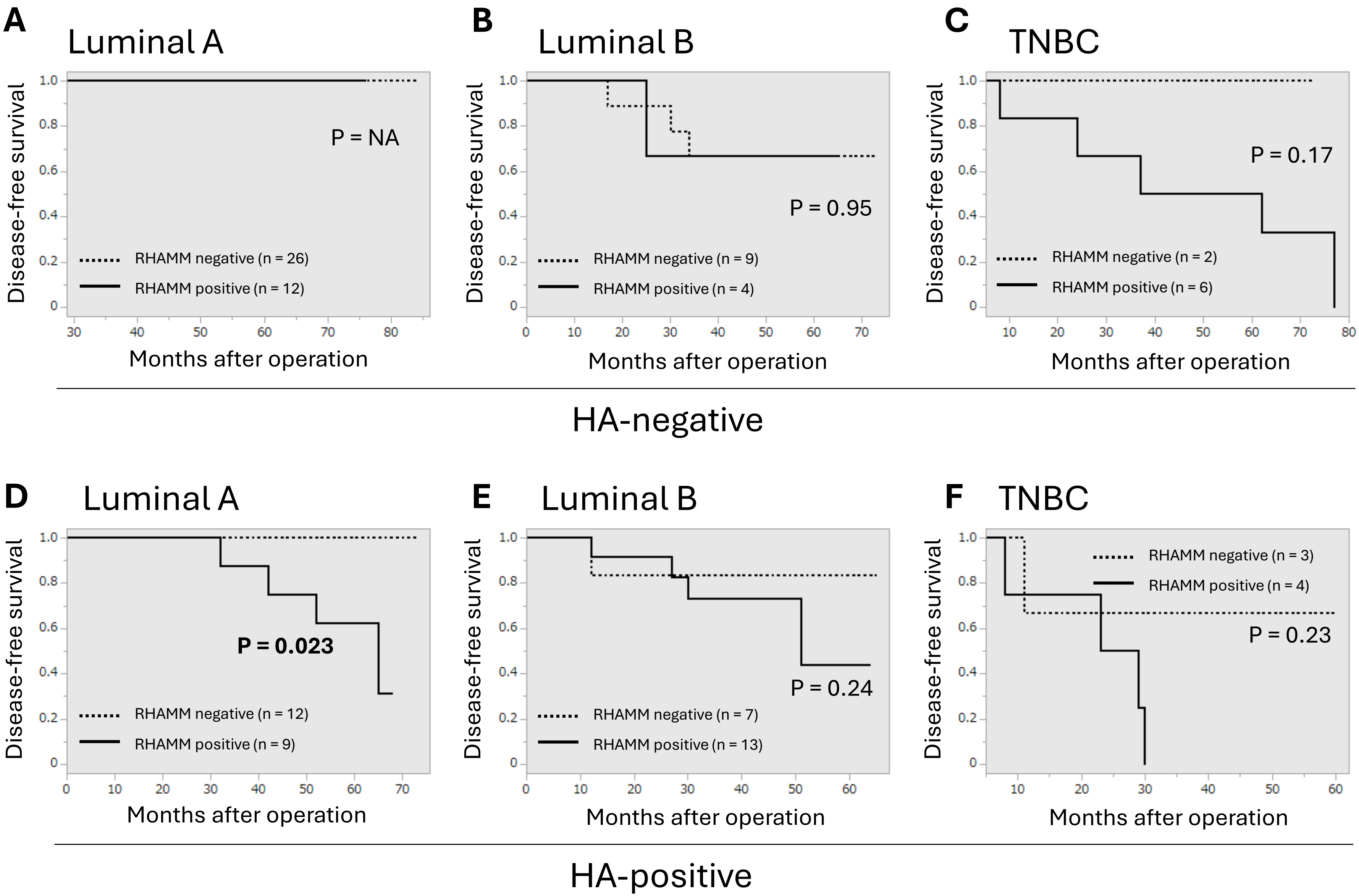

Supplementary Figure S2. Disease-free survival according to RHAMM and HA in each breast cancer subtype
Survival curves in luminal A (A, D), luminal B (B, E) and TNBC subtype (C, F) according to HA status (HA-negative; A-C, HA-positive; D-F) were generated by Kaplan-Meier method and statistical significance was assessed by log lank test.
Survival curve of HER2 type was not available because no patients had recurred in this group.

Supplementary Figure S3

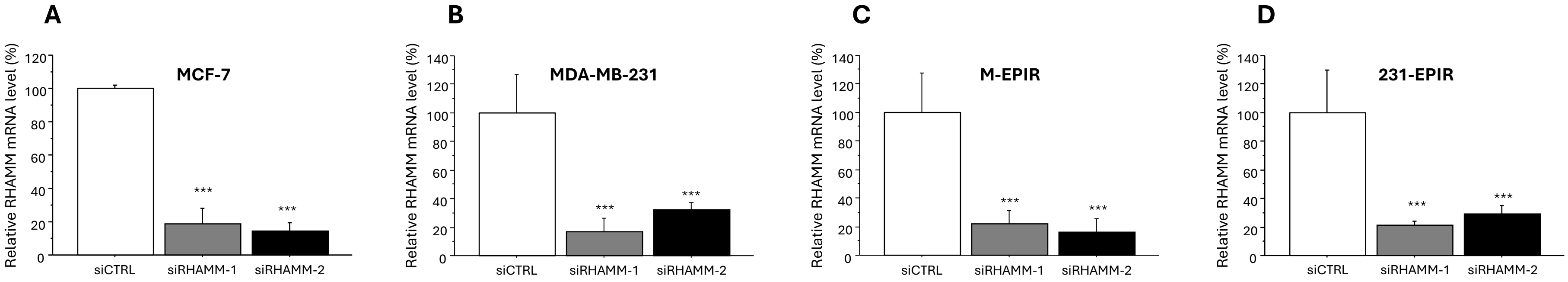

Supplementary Figure S3. Knock down efficiency of siRNA targeting RHAMM (siRHAMM-1 and siRHAMM-2)
Knock down efficiency was evaluated by real time PCR.
Both siRNAs significantly suppressed RHAMM mRNA level in MCF-7 (A), MDA-MB-231 (B), M-EPIR (C), and 231-EPIR (D).
***; P < 0.001

Supplementary Figure S4

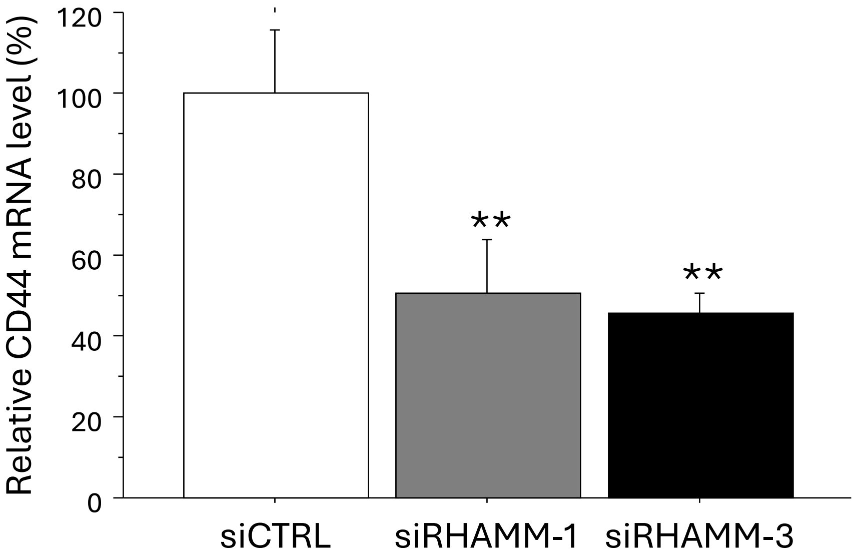

Supplementary Figure S4. Regulation of CD44 mRNA by RHAMM in T-47D. CD44 mRNA was evaluated by real time PCR. **; P < 0.01

Supplementary Table S1. The sequences of primers and siRNAs.

Primers		Sequence (5' to 3')
RHAMM	Forward	CAGCTGAAGATGAAGAAGGA
	Revserse	GCATGTAGTTGTAGCTGAAAAGG
RPL13A	Forward	CCTGGAGGAGAAGAGGAAAG
	Revserse	TTGAGGACCTCTGTGTATTT
E-cadherin	Forward	GCCTCCTGAAAAGAGAGTGGAAG
	Revserse	TGGCAGTGTCTCTCCAAATCCG
N-cadherin	Forward	ACAGTGGCCACCTACAAAGG
	Revserse	CCGAGATGGGGTTGATAATG

siRNA	Sequence (5' to 3')
siRHAMM-1	GAGCUCAAAUCAAGAAUAU
siRHAMM-2	GAAUCUGUUUGAGGAAGAA
