# Supplementary material for: Receptor for Hyaluronan Mediated Motility (RHAMM)/Hyaluronan Axis in Breast Cancer Chemoresistance
Source: Cancers (Basel). 2024 Oct 25;16(21):3600. doi: 10.3390/cancers16213600 (PMC11545538; doi:10.3390/cancers16213600)
Supplement: Supplementary file 1 [file cancers-16-03600-s001.zip › File S1. The original Western blot figures.pdf]

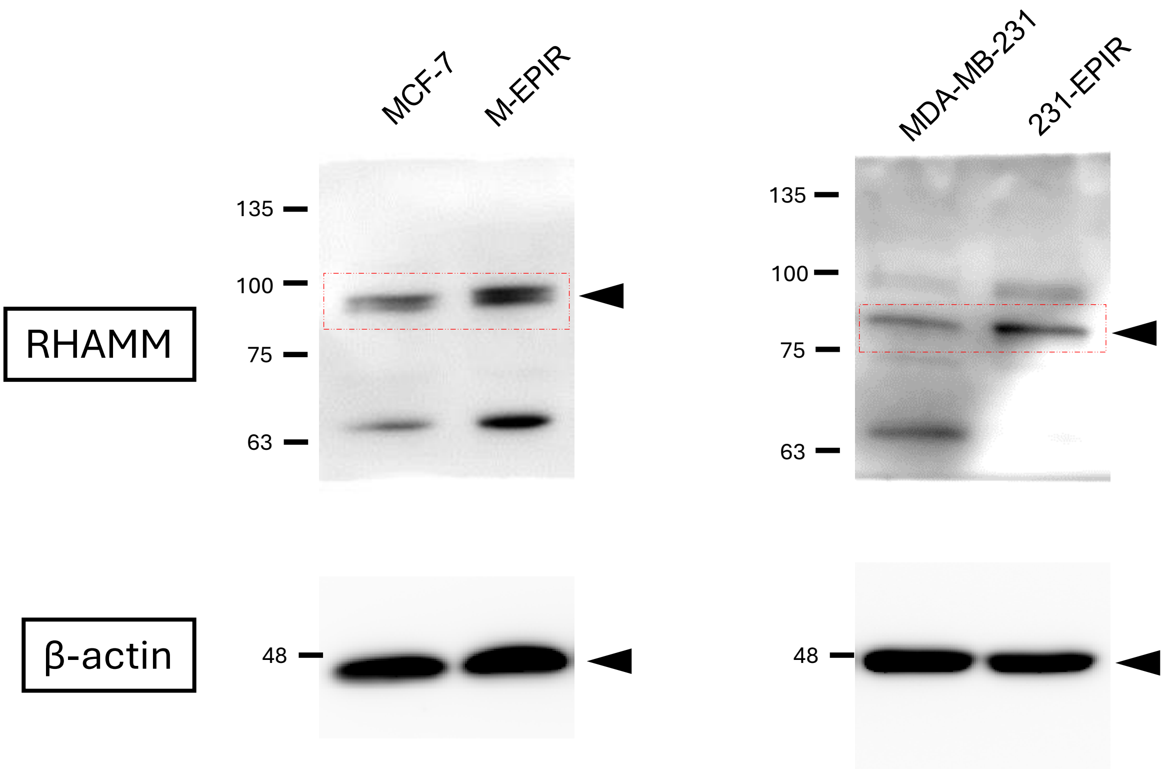

	MCF-7	M-EPIR	MDA-MB-231	231-EPIR
Densitometry readings (RHAMM)	10980.7	23680.8	6073.38	9534.96
Densitometry readings (β-actin)	36546.0	36178.5	36373.13	30639.2
RHAMM/β-actin	0.300463	0.654553	0.166974	0.311201
Fold Change	1	2.17848	1	1.86376

Supplementary materials; The whole blot and densitometry readings/intensity ratio of each band. The whole western blot showing all bands and molecular weight markers and densitometry readings/intensity ratio of each band shown in Figure 4C,D.
